# Cross-cultural validation and measurement invariance of the learning climate questionnaire in physical education: evidence from Mexico and Spain

**DOI:** 10.3389/fpsyg.2026.1706868

**Published:** 2026-02-04

**Authors:** M. Gómez-López, J. Zamarripa, D. Pizarro, R. Ramírez-Nava, D. Manzano-Sánchez

**Affiliations:** 1 Department of Physical Activity and Sport, Faculty of Sport Sciences, University of Murcia, Murcia, Spain; 2 Faculty of Sports Organization, Autonomous University of Nuevo León, Monterrey, Mexico; 3 Universidad Europea de Madrid, Faculty of Law, Education and Humanities, Department of Education and Teacher Training, Madrid, Spain; 4 Faculty of Education, Camilo José Cela University, Madrid, Spain; 5 Don Bosco Center for Higher Studies (CES Don Bosco), Complutense University of Madrid (UCM), Madrid, Spain; 6 Department of Artistic, Musical and Movement Expression, Faculty of Education, University of Murcia, Murcia, Spain

**Keywords:** learning climate, Mexican students, school, secondary education, teacher climate

## Abstract

**Introduction:**

This study examined the factorial validity and cross-cultural invariance of the Learning Climate Questionnaire in Physical Education (LCQ-EF) among adolescents from Mexico and Spain.

**Methods:**

A total of 1,753 secondary school students (896 Mexican, 857 Spanish) completed the LCQ-EF. Multigroup confirmatory factor analyses tested configural, metric, scalar, and strict invariance, including invariance across genders and two age groups (12–14 and 15–17 years).

**Results:**

The results indicated excellent internal consistency (α and ω > 0.90) in both samples and supported strict invariance between Mexico and Spain, as well as across genders and two age groups, confirming that the LCQ-EF measures autonomy support equivalently across cultures, gender, and age groups.

**Discussion:**

The findings validate the LCQ-EF as a reliable tool for cross-cultural assessment of the perceived learning climate in Physical Education. The study highlights the universality of autonomy-supportive teaching as posited by Self-Determination Theory and provides a valid instrument to evaluate teacher behaviors that foster motivation and engagement in diverse cultural contexts.

## 1 Introduction

Secondary education is a critical stage in the educational journey, bridging primary schooling and higher education. Its importance extends beyond academic learning, influencing economic growth, social development, and personal empowerment. In this sense, according to Shek et al. (2021) and Steinberg (2014), this stage involves numerous emotional, and behavioral changes. This stage fosters critical thinking, problem-solving, creativity, and a strong work ethic, preparing students for adult life and higher education (Singh, 2024; Uwaezuoke, 2022). On the other hand, adolescence is a period for personality and value development including aspects like resilience, responsibility, and emotional well-being (Evans et al., 2018) that enable adolescents to actively engage as responsible citizens and contribute to sustainable social development (Huttunen et al., 2023).

One of the most important aspects that guides human behavior is motivation. Motivation has been related to academic achievement with a positive relationship to academic performance (Cachón-Zagalaz et al., 2023) and adaptive behaviors like resilience (Supervía et al., 2019). In this sense, another important variable related to motivation is learning climate. Learning climate refers to the perceived atmosphere in a learning environment that supports or hinders students’ motivation, engagement, and learning processes. It is shaped by multiple dimensions such as autonomy support, involvement, and emotional support provided by instructors (Baeten et al., 2013; Reeve, 2016). In turn, the characteristics of the learning climate are linked to students’ motivation, as they facilitate the satisfaction of the three basic psychological needs—competence, autonomy, and relatedness—according to Self-Determination Theory (SDT; Deci and Ryan, 1985, 1991, 2000). When an adequate learning climate is provided these needs are satisfied and this promotes autonomous motivation. For this to occur, teacher engagement is essential (Reeve, 2016). Consequently, students are more inclined to develop intrinsic motivation for learning when they perceive that they have control over their educational process (autonomy), feel capable of reaching their academic objectives (competence), and experience a sense of connection with both peers and instructors (relatedness) (Formento et al., 2023).

Physical Education (PE) plays a significant role in shaping the learning climate within schools. PE classes should use student-centered teaching methods to create a more positive learning climate since these environments increase student engagement, enjoyment, and intrinsic motivation (Marcelino, 2025; Simonton et al., 2021). In this sense, teacher behaviors that provide autonomy support are linked to students’ development of life skills and psychological well-being (Cronin et al., 2018). On the other hand, learning climate focused on mastery and autonomous motivation is associated with higher satisfaction of students’ basic psychological needs, better self-determined motivation, and greater intention to participate in physical activity according to Cid et al. (2019) and Jaakkola et al. (2017). However, a performance climate focused on intragroup competition and rivalry decreases perception of autonomy, increases perception of control, is linked to less positive outcomes in PE students, and can increase amotivation (Jaakkola et al., 2017; Zheng et al., 2023).

Given the importance we have seen of motivation in the individual’s role, and specifically in students’, as well as the significant role of PE classes in their motivation, the role of the teacher, who creates an appropriate learning environment, is essential to achieving all the benefits of PE classes. Therefore, current research has sought various methods to analyze the learning environment generated by teachers in classes. Specifically, most analyses use a validated questionnaire to assess how teacher behaviors influence the climate. Existing instruments for assessing the learning climate focus on different conceptualizations grounded in distinct theoretical frameworks. For instance, the Learning and performance Orientations in PE Classes Questionnaire (LAPOPECQ) from Papaioannou (1994) has been used by several authors (e.g., Marcelino, 2025 or Rodrigues et al., 2022) and others more oriented to sport like the Perceived Motivational Climate in Sport Questionnaire (PMCSQ) by Walling et al. (1993), both based on the theoretical framework of Achievement Goal Theory (Nicholls, 1989). Apart from LAPOPECQ, the Learning Climate Questionnaire (LCQ) by Bowen and Kilmann (1975) and Williams and Deci (1996) is one of the most widely used instruments to analyze teachers’ autonomy support in PE classes within the framework of Self-Determination Theory (SDT). This questionnaire, developed by Williams and Deci (1996), has shown satisfactory results across various contexts, and specifically with secondary education students (Baena Extremera et al., 2019; Granero-Gallegos et al., 2023; Manzano-Sánchez et al., 2023). The LCQ has been adapted to other countries such as Spain (Núñez et al., 2012), Japan (Aoshima and Suzuki, 2022), and Uruguay (Vinçon et al., 2019), and some studies also address invariance across gender (Simon and Salanga, 2021).

Despite what has been observed regarding the importance of the climate created by teachers in motivation and academic outcomes, as we have seen, there are few instruments specifically adapted for PE classes, and even fewer that assess different cultural contexts. This limitation is crucial since motivation and engagement in classes are influenced by cultural and contextual factors, as specified by Self-Determination Theory (Ryan and Deci, 2000). Moreover, during secondary education, where adolescents experience significant psychological, emotional, and social changes, it becomes even more important to validate questionnaires that can be used for the evaluation of PE interventions aimed at promoting improvement and change (Blakemore and Mills, 2014). Without these culturally adapted tools, it is difficult to accurately diagnose the existing learning environment and design appropriate interventions for PE students. The LCQ has undergone various adaptations to different contexts, including a short version (Simon and Salanga, 2021; Williams and Deci, 1996), a Spanish university adaptation by Núñez et al. (2012), a PE version by Granero-Gallegos et al. (2014), or applications in youth sport settings (Bean et al., 2020), and an adaptation to the Brazilian (da Costa et al., 2016) and Mexican (Maldonado-Maldonado et al., 2017) context.

However, no studies have been found that validate the questionnaire in other cultural contexts, specifically within PE classes, apart from the study by da Costa et al. (2016), in which the age of the participants is not specified. Given the great importance of promoting and analyzing the learning climate across different cultures and contexts and considering the widespread use of this questionnaire in Spain, it is necessary to examine whether this scale is appropriate for use in other cultures. Overall, there is a clear need for culturally invariant measures of teacher autonomy support in PE. This study is grounded in Self-Determination Theory (SDT), which links autonomy-supportive learning climates to the satisfaction of students’ basic psychological needs and their motivation, engagement, and well-being. While previous national validations of the Learning Climate Questionnaire in PE, such as those by Maldonado-Maldonado et al. (2017) in Mexico and Granero-Gallegos et al. (2014) in Spain, provided evidence within specific cultural contexts, this study goes further by examining cross-cultural and multigroup measurement invariance, as well as age-specific applicability among secondary school students. By doing so, it offers new insights into the generalizability of the LCQ-EF across cultures and age groups, addressing an important gap in the literature.

Therefore, the aim of this study was to examine the validity and measurement invariance of the Learning Climate Questionnaire in the context of Physical Education (LCQ-EF) among Mexican secondary school students, in order to determine whether it is a reliable and culturally appropriate instrument for assessing perceived learning climate in this population.

## 2 Method

### 2.1 Design and participants

The present study consisted of 1,753 PE students from two different cultural contexts: Mexico and Spain. The Mexican sample was composed of 896 students (after removing 4 cases with missing data from an initial pool of 900), including 463 boys and 433 girls, aged between 12 and 17 years (*M* = 13.62; SD = 0.997), from 12 public (80%) and 3 private (20%) schools in the metropolitan area of Monterrey, Mexico. In turn, the Spanish sample included 857 students aged 12 to 18 years (boys = 436; girls = 421; *M* = 14.298; SD = 1.475) enrolled in different educational institutions in the Region of Murcia. Given the minimal proportion of missingness in the Mexican sample (<0.5%), listwise deletion was applied; no imputation procedures were used.

### 2.2 Instruments

Support for autonomy, the Spanish version adapted to PE (LCQ-EF) by Granero-Gallegos et al. (2014) was used, which comes from the Learning Climate Questionnaire (LCQ; Williams and Deci, 1996, adapted to Spanish context by Núñez et al., 2012). The questionnaire consists of 14 items to measure the support for autonomy by the teacher, through a dimension called: support for autonomy. In the instructions, the subjects are asked to indicate the degree of agreement with the items. The answers were collected on a 7-point polytomic item scale ranging from 1 (strongly disagree) to 7 (strongly agree). This scale showed a high internal consistency *α* = 0.963. Example items include, for the Mexican version: “*En esta clase de Educación Física sentimos que el maestro nos da opciones y posibilidades*” and for the Spanish version: “*Siento que mi profesor/a de EF me ofrece distintas alternativas y opciones*”.

### 2.3 Procedure

Prior to conducting the fieldwork, formal authorization was obtained from the school management, the School Council, and the PE teachers of the selected courses, as well as informed consent from parents. Parents received a letter outlining the study objectives, procedures, and a copy of the instrument. Before initiating data collection, interviewers received specific training following the guidelines of Manzano et al. (1996). This training addressed strategies for presenting the project to students and faculty, familiarization with the questionnaire items, pair-based practice, and discussion of potential student queries or reactions. Interviewers were additionally trained in the use of field logs, data recording protocols, and methods to enhance response rates and minimize bias. A pilot test was subsequently conducted with a comparable sample to identify potential design limitations.

Students were informed about the purpose of the study, its voluntary nature, confidentiality of responses, and the absence of right or wrong answers, and were encouraged to respond with complete honesty. The instrument was self-administered during a regular school day, under the supervision of two trained interviewers and the PE teacher. Administration required approximately 20 min of class time, depending on student age. Only participants with prior parental consent were included. Ethical approval was granted by the Research Ethics Committee of the University of Murcia (ID: 4447/2023).

### 2.4 Data analysis

The factorial equivalence of the LCQ-EF between Mexican and Spanish students was assessed through a multigroup factorial invariance analysis. The process was carried out hierarchically, beginning with the evaluation of configural invariance, followed by metric, scalar, and strict invariance, in line with the procedure recommended by Wang and Wang (2012).

Model comparisons were conducted using the fit indices RMSEA (Root Mean Square Error of Approximation), CFI (Comparative Fit Index), and NNFI (Non-Normed Fit Index). To consider invariance as being maintained when moving from a less restrictive to a more restrictive model, the following cutoff criteria were used: differences in CFI (ΔCFI) and NNFI (ΔNNFI) values less than or equal to 0.01, and differences in RMSEA (ΔRMSEA) values less than or equal to 0.015 (Cheung and Rensvold, 2002; Wang and Wang, 2012).

Following the recommendations of Li (2016) and Rogers (2024), all analyses were performed using the Diagonally Weighted Least Squares (DWLS) estimator, which is particularly suitable for ordinal data (such as responses on Likert-type scales), when there is a reasonably large sample size and when there are violations of normality assumptions. For this purpose, the software JASP version 0.18.3 (JASP Team, 2024) was employed.

## 3 Results

### 3.1 Preliminary analysis

The descriptive results factor loadings, and item–total correlations of the LCQ-EF in both samples are presented in Table 1. The means and standard deviations of the items show similar patterns between Mexican and Spanish students, suggesting consistency in the perception of the construct under evaluation across both contexts.

**Table 1:** Descriptives, skewness, kurtosis, factor loading, and item-rest correlation of the LCQ-EF items in Mexico and Spain.

Item	Mexico (*n* = 896)						Spain (*n* = 857)					
	*M*	SD	*S*	*K*	FL	*r*_(it–rest)_	*M*	SD	*S*	*K*	*FL*	*r*_(it–rest)_
1	4.831	1.960	−0.681	−0.773	0.621	0.575	4.646	1.933	−0.371	−0.958	0.694	0.625
2	5.073	1.808	−0.867	−0.297	0.713	0.666	4.897	1.839	−0.487	−0.856	0.833	0.777
3	5.613	1.734	−1.280	0.625	0.789	0.696	5.279	1.745	−0.753	−0.434	0.839	0.763
4	5.539	1.584	−1.200	0.692	0.731	0.690	5.340	1.711	−0.804	−0.342	0.856	0.775
5	5.932	1.776	−1.724	1.742	0.445	0.370	5.546	1.714	−1.055	0.142	0.842	0.744
6	5.631	1.663	−1.360	1.016	0.664	0.618	5.351	1.734	−0.860	−0.209	0.743	0.660
7	4.390	1.932	−0.378	−1.065	0.671	0.639	5.242	1.709	−0.711	−0.474	0.784	0.714
8	5.142	1.861	−0.875	−0.312	0.817	0.744	5.047	1.880	−0.661	−0.660	0.797	0.750
9	5.637	1.728	−1.342	0.850	0.673	0.592	5.278	1.715	−0.776	−0.346	0.805	0.744
10	4.854	1.919	−0.637	−0.782	0.714	0.662	4.676	1.859	−0.424	−0.856	0.816	0.761
11	4.886	1.733	−0.719	−0.413	0.784	0.736	4.770	1.834	−0.422	−0.823	0.824	0.761
12	5.223	1.732	−0.924	−0.061	0.748	0.689	4.868	1.828	−0.477	−0.860	0.847	0.786
13	5.070	1.685	−0.852	−0.141	0.727	0.685	4.732	1.851	−0.429	−0.839	0.825	0.770
14	3.906	2.082	−0.067	−1.357	0.709	0.633	4.134	2.144	−0.079	−1.350	0.698	0.620

*n* = sample, *M* = mean, SD = standard deviation, *S* = skewness, *K* = kurtosis, FL = factor loading, *r*_(it–rest)_ = item-rest correlation.

Regarding the reliability of the scale, the results were satisfactory. For the Mexican sample, Cronbach’s alpha reached a value of 0.919, McDonald’s omega was 0.924, and AVE (Average Variance Extracted) was 0.496. Similarly, in the Spanish sample, even higher values were obtained, with an alpha of 0.948, an omega of 0.969, and an AVE of 0.643, indicating excellent internal consistency in both samples.

### 3.2 Results of the multigroup factorial invariance analysis

The equivalence of the instrument in Mexican and Spanish students was tested through a series of factorial invariance analyses using the DWLS method. Table 2 presents the main fit indices for each of the evaluated models.

**Table 2:** Fit indices of the measurement invariance models for the LCQ-EF between Mexico and Spain.

Model description		χ^2^	df	RMSEA (90% IC)	CFI	NNFI	SRMR	∆RMSEA	∆CFI	∆NNFI
M0a	Base model Mexico	232.850**	76	0.048 (0.041–0.055)	0.996	0.995	0.038			
M0b	Base model Spain	554.067**	76	0.086 (0.079–0.092)	0.995	0.994	0.045			
M1	Structural invariance (base model)	786.917**	152	0.069 (0.064–0.074)	0.995	0.995	0.041			
M2	Invariance FL	1,445.832**	165	0.094 (0.090–0.099)	0.991	0.990	0.063	0.002	0.004	0.005
M3	Invariance FL + INT	1,868.349**	234	0.089 (0.086–0.093)	0.988	0.991	0.045	0.002	0.007	0.004
M4	Invariance FL + Int. + Error	1,868.349**	235	0.089 (0.085–0.093)	0.988	0.991	0.045	0.002	0.007	0.004

df = degrees of freedom; RMSEA = root mean square error of approximation; 90% CI = 90% confidence interval for RMSEA; NNFI = non-normed fit index; CFI = comparative fit index; SRMR = standardized root mean square residual; FL = factor loadings; Int. = intercepts. All comparisons in the Δ indices are made with respect to the model (M1); **p < 0.01.

First, the baseline model for Mexico (M0a) was analyzed, which showed excellent fit indices, indicating that the factorial structure adequately fit the Mexican sample data. Similarly, the baseline model for Spain (M0b) also revealed acceptable fit indices, suggesting that the structure is equally valid for Spanish students (see Table 2).

Subsequently, multisample analyses were conducted by creating new nested models. Model 1, which examined the structural invariance of the instrument across both groups (same factorial structure without constraints), showed a good fit (RMSEA = 0.069, CFI and NNFI = 0.995), suggesting that the general factorial structure is shared between both groups.

In the next step, factorial loadings invariance was evaluated (M2, FL invariance). Here, RMSEA increased, and CFI and NNFI slightly decreased (see Table 2). However, the change in CFI relative to the baseline model (M1) remained below the critical threshold of 0.01, indicating that imposing equality of factor loadings across groups does not significantly affect model fit. In other words, the items appear to relate to the factors in a similar way in both countries.

Next, invariance of loadings and intercepts was tested (M3, FL + INT invariance). RMSEA remained stable, NNFI showed a slight increase, but CFI dropped to 0.988. Nevertheless, the change in CFI remained small (ΔCFI = 0.007). This suggests that, in addition to the loadings, the item intercepts can also be considered equivalent across groups, allowing for direct comparisons of latent means.

Finally, the most restrictive model was evaluated (M4, invariance of loadings, intercepts, and errors). The fit indices showed virtually no changes compared to the previous model, indicating that even when error variances were constrained, the overall model fit was not significantly compromised.

Similarly, factorial invariance of the LCQ-EF was examined according to students’ sex. Table 3 presents the results of the multigroup confirmatory factor analysis (CFA), where the two baseline models (M0a for males and M0b for females) showed a good fit to the data in both samples (see Table 3). These findings indicate that the model performs adequately for both male and female students.

**Table 3:** Fit indices of the multigroup factorial invariance models by sex.

Model description		χ^2^	df	RMSEA (90% IC)	CFI	NNFI	SRMR	∆RMSEA	∆CFI	∆NNFI
M0a	Base model male	260.828**	76	0.052 (0.045–0.059)	0.997	0.996	0.037			
M0b	Base model female	342.250**	76	0.064 (0.057–0.071)	0.996	0.995	0.041			
M1	Structural invariance (base model)	603.079**	152	0.058 (0.053–0.063)	0.996	0.996	0.039			
M2	Invariance FL	721.007**	165	0.062 (0.057–0.067)	0.995	0.995	0.042	0.004	0.001	0.001
M3	Invariance FL + INT	784.361**	234	0.052 (0.048–0.056)	0.995	0.996	0.039	0.006	0.001	0.000
M4	Invariance FL + Int. + Error	785.365**	235	0.052 (0.048–0.056)	0.995	0.996	0.039	0.006	0.001	0.000

df = degrees of freedom; RMSEA = root mean square error of approximation; 90% CI = 90% confidence interval for RMSEA; NNFI = non-normed fit index; CFI = comparative fit index; SRMR = standardized root mean square residual; FL = factor loadings; Int. = intercepts. All comparisons in the Δ indices are made with respect to the model (M1); ** *p* < 0.01.

The results of the configural model (M1) showed that the underlying structure of the construct was equivalent across sexes. After constraining the factor loadings to be equal (M2, metric invariance), the differences in fit indices were minimal compared to M1, supporting the equivalence of item–factor relationships between men and women. When intercepts were also constrained to be equal (M3, scalar invariance), the model fit improved slightly in RMSEA, with these changes being trivial relative to M1. This finding supports scalar invariance, allowing for meaningful comparisons of latent means across sex. Finally, after constraining the residuals (M4, strict invariance), the model fit remained virtually identical to M3, and the differences relative to M1 were negligible. This suggests that equality of residual variances does not affect model fit and provides further evidence supporting strict invariance between male and female students.

Age-related factorial invariance of the LCQ-EF was also examined. Table 4 presents the results of the multigroup confirmatory factor analysis. Given the absence of 18-year-old students in the Mexican sample and the very small representation of this age (three cases) in the Spanish sample, age groups were defined as follows: students aged 12–14 years and students aged 15–17 years. The two baseline models (M0a for the 12–14-year-old group and M0b for the 15–17-year-old group) showed good fit to the data in both age groups (CFI ≥ 0.996, NNFI ≥ 0.995, RMSEA ≤ 0.077; see Table 4), indicating that the model adequately reproduces the construct structure in both younger and older students.

**Table 4:** Fit indices of the multigroup factorial invariance models by age groups.

Model description		χ^2^	df	RMSEA (90% IC)	CFI	NNFI	SRMR	∆RMSEA	∆CFI	∆NNFI
M0a	Base model 12–14 years	199.699**	76	0.051 (0.045–0.057)	0.997	0.996	0.036			
M0b	Base model 15–17 years	355.915**	76	0.077 (0.069–0.077)	0.996	0.995	0.045			
M1	Structural invariance (base model)	655.615**	152	0.062 (0.057–0.066)	0.996	0.995	0.040			
M2	Invariance FL	729.346**	165	0.063 (0.058–0.067)	0.996	0.995	0.042	0.001	0.000	0.000
M3	Invariance FL + INT	837.092**	234	0.054 (0.050–0.058)	0.995	0.996	0.040	0.008	0.001	0.001
M4	Invariance FL + Int. + Error	837.640**	235	0.054 (0.050–0.058)	0.995	0.996	0.040	0.008	0.001	0.001

df = degrees of freedom; RMSEA = root mean square error of approximation; 90% CI = 90% confidence interval for RMSEA; NNFI = non-normed fit index; CFI = comparative fit index; SRMR = standardized root mean square residual; FL = factor loadings; Int. = intercepts. All comparisons in the Δ indices are made with respect to the model (M1); ** *p* < 0.01.

The configural model (M1) showed satisfactory fit (RMSEA = 0.062, CFI = 0.996, NNFI = 0.995), indicating that the underlying structure of the LCQ-EF is equivalent across age groups. When factor loadings were constrained to be equal (M2, metric invariance), changes in fit indices relative to M1 were minimal (ΔCFI = 0.000, ΔNNFI = 0.000, ΔRMSEA = 0.001), supporting the equivalence of item–factor relationships between the 12–14 and 15–17 age groups. When intercepts were additionally constrained (M3, scalar invariance), model fit even improved in terms of RMSEA, and changes in fit indices relative to M1 remained trivial (ΔCFI = 0.001, ΔNNFI = 0.001). This finding supports scalar invariance and allows for meaningful comparisons of latent means across age groups.

Finally, when residual variances were also constrained (M4, strict invariance), model fit remained virtually identical to M3 (RMSEA = 0.054, CFI = 0.995, NNFI = 0.996), and the differences relative to M1 were negligible. Taken together, these results provide evidence of configural, metric, scalar, and strict invariance of the LCQ-EF between students aged 12–14 and 15–17 years, supporting the comparability of latent scores obtained with the instrument across these age groups.

## 4 Discussion

The aim of this study was to analyze the factorial validity and cross-cultural invariance of the Learning Climate Questionnaire in Physical Education (LCQ-EF) among Mexican and Spanish adolescents. The results confirmed excellent internal consistency in both samples and demonstrated configural, metric, and scalar invariance across groups, supporting the adequacy of the LCQ-EF to measure students’ perceptions of autonomy support in culturally diverse settings.

At the global level, the internal consistency indices (Cronbach’s *α* and McDonald’s *ω* above 0.90) were excellent, replicating previous findings in Spanish students (Granero-Gallegos et al., 2014) and aligning with other adaptations in Brazil (da Costa et al., 2016) and Italy (Monacis et al., 2023). The confirmatory factor analyses revealed acceptable fit indices in both cultural samples. Although most factor loadings were high, some items showed slightly lower standardized loadings in the Mexican sample, suggesting that subtle contextual or linguistic nuances may influence how certain statements are interpreted. These lower loadings may reflect cultural differences in how students perceive the role of the teacher and the degree of autonomy typically allowed during Physical Education classes. In some educational contexts, teachers tend to adopt a more directive role, which may lead students to be less familiar with expressing preferences or selecting among different activity options. Thus, items referring to choice or negotiation may be interpreted differently depending on these classroom norms. Similar patterns have been reported in other LCQ adaptations, where certain items required wording adjustments or showed weaker psychometric behavior (Núñez et al., 2012; Monacis et al., 2023). Therefore, these differences seem to reflect contextual norms rather than limitations of the scale.

The multigroup analysis confirmed configural, metric, scalar, and strict invariance, indicating that the LCQ-EF operates equivalently across Mexican and Spanish adolescents. According to Cheung and Rensvold (2002), achieving metric and scalar invariance allows researchers to validly compare associations between variables and latent mean differences across groups. Practically, metric invariance ensures that items relate to autonomy support in the same way across countries, allowing comparisons of relationships with outcomes such as engagement or motivation. Scalar invariance permits meaningful comparisons of latent means, ensuring that differences reflect true perceptions rather than measurement bias. Importantly, strict invariance was also supported, meaning that residual variances are equivalent between groups (Wang and Wang, 2012). This level of invariance strengthens confidence that observed score differences are due to actual differences in perceived autonomy support, not to systematic measurement error, making the LCQ-EF particularly robust for cross-cultural research and educational evaluation.

In addition to gender invariance, this study also tested and confirmed measurement invariance across two adolescent age groups (12–14 and 15–17 years). The LCQ-EF demonstrated configural, metric, scalar, and strict invariance between these groups, indicating that the questionnaire assesses perceived autonomy support equivalently across early and middle adolescence. These results support the age-specific applicability of the scale and permit valid comparisons of latent means across developmental stages. It should be noted, however, that age groups were restricted to 12–17 years due to the absence of 18-year-old students in the Mexican sample and the minimal representation of this age in the Spanish sample; therefore, results should not be generalized to students younger than 12 or aged 18 and older.

These findings support the universality postulated by Self-Determination Theory (Deci and Ryan, 2000; Ryan and Deci, 2017). Autonomy-supportive climates were perceived consistently across Mexican and Spanish adolescents, reinforcing the idea that the basic psychological needs of autonomy, competence, and relatedness are cross-cultural in nature. In line with previous studies (Jaakkola et al., 2017; Cid et al., 2019), the validation of the LCQ-EF provides a reliable instrument to evaluate teacher behaviors that promote intrinsic motivation, engagement, and participation in PE. Given that adolescence is a critical stage for motivational development (Blakemore and Mills, 2014; Evans et al., 2018), having culturally invariant tools allows researchers and practitioners to assess the impact of interventions across diverse populations.

From a practical perspective, the LCQ-EF enables teachers and schools to evaluate how their behaviors foster autonomy support in PE. Previous evidence suggests that such environments increase enjoyment, life skills, and long-term adherence to physical activity (Cronin et al., 2018; Formento et al., 2023). The present findings make it possible to design and compare pedagogical programs in different countries, with the confidence that the tool measures the same underlying construct.

Some limitations should be acknowledged. First, although reliability and validity indices were robust, a small number of items showed comparatively lower factor loadings in the Mexican sample. This may reflect minor linguistic or contextual nuances in item interpretation, and future studies might explore whether additional wording refinements further improve the psychometric performance of these items across cultural groups. Second, the study was limited to two Spanish-speaking populations (Mexico and Spain), which may restrict the generalization of the results to other cultures and linguistic contexts. Future research may extend these analyses to additional cultural contexts or educational levels to further confirm the generalizability of the LCQ-EF.

## 5 Conclusion

This study provides strong evidence for the factorial validity and cross-cultural measurement invariance of the Learning Climate Questionnaire in Physical Education (LCQ-EF) among adolescents from Mexico and Spain. The findings confirmed configural, metric, scalar, and strict invariance of the LCQ-EF, demonstrating that the instrument measures perceived autonomy support equivalently across groups. In addition, the LCQ-EF demonstrated full measurement invariance across two adolescent age groups (12–14 and 15–17 years), supporting its use for age-related comparisons within secondary education. The analyses revealed strict measurement invariance across genders, confirming that the LCQ-EF is equally applicable to both male and female adolescents. This validation currently applies specifically to adolescents in Physical Education classes in Mexico and Spain, and generalization to other populations or educational contexts should be made with caution.

From a theoretical perspective, the results reinforce the universality postulated by Self-Determination Theory, highlighting that autonomy support is a core element of the learning climate in PE regardless of cultural background. Practically, the LCQ-EF emerges as a valid tool for teachers and researchers to assess the motivational quality of PE classes and to design programs that foster intrinsic motivation and long-term engagement in physical activity.

Future studies should extend the validation of the LCQ-EF to other cultural contexts and examine its longitudinal stability, assessing whether the instrument is sensitive to changes resulting from teacher training or pedagogical interventions.

## Data Availability

The datasets generated and analyzed during the current study are available upon request. Interested researchers may obtain access by contacting the corresponding author at the following email address: jorge.zamarriparv@uanl.edu.mx.
